# Consensus recommendations for dementia risk reduction policy: Insights from a multidisciplinary modified‐Delphi process

**DOI:** 10.1002/alz70860_099602

**Published:** 2025-12-23

**Authors:** Harriet Demnitz‐King

**Affiliations:** ^1^ Queen Mary University of London, London, United Kingdom

## Abstract

**Background:**

In the absence of a cure or widely accessible, effective treatments, prevention efforts are critical to mitigating the future burden of dementia. Achieving this requires coordinated efforts across health and social care systems, alongside engagement from other government sectors and key stakeholders. However, translating evidence into effective and equitable policies presents a significant challenge. To address this, the NIHR Dementia and Neurodegeneration Policy Research Unit at Queen Mary University of London (DeNPRU‐QM) launched an expert consensus initiative aimed at developing actionable public health policy recommendations for dementia prevention.

**Method:**

The study was pre‐registered on Open Science Framework (https://osf.io/5c4mh). A diverse, multidisciplinary panel of 40 experts balanced across lived, academic, clinical, policy and advocacy experience, career stages, gender and ethnicity met in October 2024 at Nottingham University for a two‐day in‐person workshop. They shared perspectives regarding how public health policy should respond to/account for current dementia prevention research, co‐creating preliminary statements to reflect views discussed and receiving support across stakeholder groups. This was followed by a three‐round online modified‐Delphi survey where panellists rated statements on a 5‐point Likert scale, provided feedback, and suggested new statements. In rounds 2 and 3, statements were revised based on feedback, and panellists could adjust ratings after reviewing group comments and prior results. Consensus was defined as ≥67% agreement, with statements achieving this threshold graded as ‘U’ (100% agreement), ‘A’ (90%–99% agreement), ‘B’ (78%–89% agreement), or ‘C’ (67%–77% agreement).

**Result:**

38 (95%) panellists completed all three survey rounds. Consensus was achieved on 56 recommendations spanning four domains: public health messaging, individual‐level interventions, population‐level interventions, and research commissioning. Six statements achieved unanimous agreement; these highlighted the need for consistent, evidence‐based messaging, equitable access to interventions, systemic approaches to reduce health disparities, and improved data infrastructure for tracking dementia outcomes.

**Conclusion:**

This framework provides a robust foundation for policymakers seeking to implement evidence‐based dementia prevention. By prioritising clear communication, targeted interventions, and sustained investment in research, the recommendations can guide efforts to address structural inequities and advance dementia risk reduction. Ongoing cross‐sector advocacy will be crucial to drive policy adoption and implementation.

